# Executive Functions and Emotion–Attention Interaction in Assessment of Brain Health: Reliability of Repeated Testing With Executive RT Test and Correlation With BRIEF-A Questionnaire

**DOI:** 10.3389/fpsyg.2018.02556

**Published:** 2018-12-11

**Authors:** Mikko Erkkilä, Jari Peräkylä, Kaisa M. Hartikainen

**Affiliations:** ^1^Behavioral Neurology Research Unit, Tampere University Hospital, Tampere, Finland; ^2^Faculty of Medicine and Life Sciences, University of Tampere, Tampere, Finland

**Keywords:** executive functions, emotion, assessment, learning, practice effect, test–retest reliability, go/no-go, brain health

## Abstract

Executive functions (EF) rely on intact fronto-subcortical networks. An insult, disorder or treatment compromising brain health may impair the functioning of these widespread networks and consequently disrupt EF. Changes in brain health due to treatment or disorder can be assessed by repeating an EF test at different time points, but practice effect may confound the results. In this study we examined reliability of repeated testing using a computer-based test of EF, Executive Reaction Time (RT) Test, that allows assessment of different executive functions and emotion–attention interaction. In addition, we investigated whether performance measures correlate with scores derived from a clinically validated questionnaire of executive functions, Behavior Rating Inventory of Executive Function, Adult version (BRIEF-A). Healthy subjects performed the test twice, 3–4 weeks apart. When the entire tests were compared, subjects were faster and their odds to make an error reflecting disruption of working memory was lower in the second test. When two (error analysis) or four (RT analysis) blocks out of total eight test blocks were removed from the beginning of the test, the differences disappeared. In the first test emotional distractors prolonged RTs of younger, but not older, participants. In the second test emotional distractors had no effect on RTs of either age group. RTs correlated with Global Executive Composite score of BRIEF-A. Test–retest reliability analysis showed that the Executive RT Test is reliable in repeated testing with 0.83 intraclass correlation coefficient for RTs, 0.72 for total errors and 0.68 for working memory related errors. In summary, performance speed in the Executive RT Test correlate with subjective evaluations of executive functions and is reliable in repeated assessment when enough practice is ensured before the actual test. Thus, the Executive RT test holds promise as a potential indicator of brain health reflecting level of executive functions linked with daily life demands as well as typical emotion–attention interaction or possible aberrations in it.

## Introduction

Executive functions are higher level cognitive control processes involved in setting goals, planning strategies and monitoring one’s activities to achieve those goals (Jurado and Rosselli, 2007). Large brain regions and widespread brain circuits including prefrontal cortex and its networks subserve executive functions and consequently executive functions are vulnerable to different brain disorders, conditions and insults that directly or indirectly compromise the functioning of this distributed network. Accurate assessment of executive functions is crucial not only because executive functions are critical for everyday life and independent living, but because executive functions reflect brain health in general (Lezak, 1982; Diamond, 2013; Jacobs et al., 2013).

While there are many patients with deficits in executive functions presenting with challenges in their everyday life, problems frequently remain undetected with widely used conventional neuropsychological tests (Verdejo-García and Pérez-García, 2007; Løvstad et al., 2012). Conventional neuropsychological tests tend to focus on isolated cognitive processes rather than co-operation and integration of several cognitive processes (Alvarez and Emory, 2006) and they are conducted in structured testing environments as opposed to distractible, unpredictable and unstructured real-world environments with parallel demands on multiple cognitive processes. Furthermore, in contrast to testing environments that are typically emotionally neutral or supportive, in real-world environments emotional challenges interact with executive functions (Chaytor and Schmitter-Edgecombe, 2003; Kuusinen et al., 2018). Thus, neuropsychological tests are conducted in ideal environments for optimal cognitive performance in contrast to unideal real-world environments that challenge executive functions to a greater extent in everyday life. These differences in testing and real-world environments contribute to compromised ecological validity of neuropsychological tests of executive functions (Sbordone, 2001). Consequently, it is not surprising that conventional neuropsychological test may fail to detect some of the everyday life challenges in executive functions patients encounter (Shallice and Burgess, 1991; Hanna-Pladdy, 2007). To that end, there is a tremendous need for experimental studies on novel ways to assess executive functions that account for some of the above-mentioned challenges.

Another challenge with conventional neuropsychological tests in a clinical setting is the need for repeated testing either to be able to assess the progress of a disorder or the efficacy of rehabilitation or a treatment on executive functions. However, typically performance in tests of executive functions improve with repetition (Bartels et al., 2010), making it hard to evaluate whether the improvement in performance is due to improved brain health along with truly improved level of executive functions or merely due to repeated testing. Even rather long testing intervals, such as 1 year between the tests, may lead to improved results in tests requiring inhibition and mental flexibility due to practice (Haatveit et al., 2015).

A common way to examine the test repeatability and reliability is a test–retest correlation. Test–retest correlations are low especially in the tests of executive functions (Lowe and Rabbitt, 1998). Executive functions are characteristically needed in novel situations and tests of executive functions are designed to be novel (Lowe and Rabbitt, 1998), but when the same test is repeated, the test is no longer novel. Using tests with significant practice effect and low test–retest reliability the assessment of the efficacy of an intervention, such as treatment or rehabilitation, in patients with impaired executive functions, is challenging (Lemay et al., 2004).

The Executive RT Test is a computer-based test of executive functions designed to overcome some of the limitations of the conventional neuropsychological tests by mimicking everyday life demands on cognitive control functions with multiple executive functions engaged simultaneously and in context of task-irrelevant threat related emotional stimuli (Hartikainen et al., 2010). The Executive RT Test engages the frontal circuits diversely by challenging attention, working memory, inhibition, set shifting and emotional control simultaneously.

The test has previously been shown to be sensitive in detecting mild alterations in executive functions, both impairment in patients with persistent symptoms after mild traumatic brain injury (Hartikainen et al., 2010) and improvement in patients having undergone aortic valve replacement surgery due to aortic stenosis (Liimatainen et al., 2016). It has also been used to study the impact of neuromodulation such as deep brain stimulation (Hartikainen et al., 2014) and vagus nerve stimulation (Sun et al., 2017) on affective and cognitive functions and specifically executive functions in patients with refractory epilepsy, as well as the roles of different brain regions such as the orbitofrontal cortex (Mäki-Marttunen et al., 2017), the anterior nucleus of the thalamus (Hartikainen et al., 2014; Sun et al., 2015) and the mediodorsal nucleus of the thalamus (Peräkylä et al., 2017) in these functions.

Sensitive, repeatable tests that objectively reflect subjective challenges in executive functions are needed not only to detect problems in patients with different brain disorders or damage but also to conduct intervention studies that allow for identifying factors that contribute to improved brain health. While the Executive RT Test has shown promise as a sensitive method for detecting subtle alterations in executive functions and it has been successfully used in number of different patient groups, no previous study has looked at the impact of repeated testing with the Executive RT Test. The main aim of the current study was to investigate the reliability of repeated testing of executive functions with the Executive RT Test using objective performance measures. Another aim was to study the impact of practice on task performance, as well as potential changes in emotion–attention interaction as measured by emotional interference in performance in repeated tests. We also assessed whether age has an impact on any of the observed effect. The two most common test–retest correlation measures, Pearson’s correlation coefficient and intra-class correlation (ICC) coefficient, were calculated to allow comparison of the Executive RT Test with other commonly used executive tasks. The Executive RT Test allows recording event-related potentials along with cognitive performance providing means to assess brain health simultaneously with physiological and behavioral measures.

In addition we studied the repeated assessment of executive functions using clinically validated questionnaire reflecting subjective problems of executive functions in everyday life, the Behavior Rating Inventory of Executive Functions-Adult version (BRIEF-A) (Roth et al., 2005). The use of the BRIEF-A questionnaire gave us a reference point to which we were able to compare test–retest reliability of the Executive RT Test. Furthermore, we correlated scores derived from the BRIEF-A with performance measures of the Executive RT Test to assess whether subjective evaluation of executive functions in daily life correlate with objective measures in a computer-based test of executive functions.

## Materials and Methods

### Subjects

Twenty healthy subjects (mean age 37.1 years, sd 12.1 years, min age 21 years, max age 60 years, 10 males and 10 females) selected with convenience sampling method participated in the study. Subjects conducted the Executive RT Test and filled in Behavioral Inventory of Executive Functions, Adult version (BRIEF-A) questionnaire twice, 3–4 weeks apart. Exclusion criteria were any neurological or psychiatric disease history. The study was approved by the Regional Ethical Committee of Tampere University Hospital, Tampere, Finland and at the beginning of the first session all subjects gave written informed consent according to the guidelines set forth in the Declaration of Helsinki governing the treatment of human subjects.

### Executive RT Test

**FIGURE 1:**
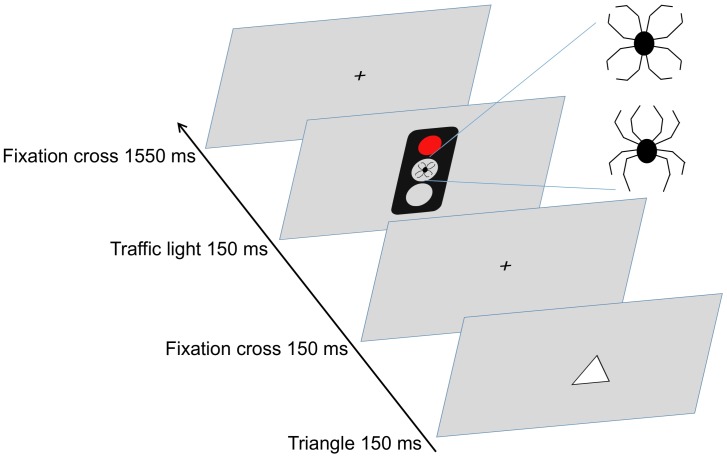
The triangle is presented in the middle of the screen for 150 ms. The color of the traffic light indicates whether the subject is required to press (Go signal) one of the two buttons according to the orientation of the triangle or to withhold from responding (No-go signal). Emotionally neutral or threatening distractor is shown in the centermost circle of the traffic light.

The Executive Reaction Time (RT) Test is a Go/No-go test tapping into working memory, response inhibition, emotional interference and task switching (Hartikainen et al., 2010). In the Executive RT Test subject is required to respond to a visual stimulus, a triangle pointing up or down, by pressing one of the two keys on the response pad according to the orientation of the triangle (Go condition) or by not responding at all (No-go condition). The orientation of the triangle is randomized within each block. Go/No-go condition i.e., whether the subjects should respond or withhold from responding, is indicated by a green or a red traffic light. The rule for responding changes between each block i.e., whether green or red light indicates a Go condition and vice versa. There were four Green-Go Red-No-go and four Red-Go Green No-go blocks, totaling into 512 trials. In the centermost circle of the traffic light there is an emotional distractor i.e., black line drawing of a spider, a biologically relevant threat stimulus (Öhman et al., 2001), or an emotionally neutral control figure composed of the exact same line elements but in a different configuration resembling a flower. A trial begins with a triangle presented in the middle of the screen for 150 ms, followed by the fixation cross for 150 ms and by the traffic light 150 ms. One trial last approximately 2000 ms and subject has approximately 1550 ms to respond. There is a 150 ms jitter associated with the onset of the trial to prevent subjects from synchronizing their responding to the rhythm of stimulus presentation (Figure 1).

The Executive RT Test performance measures include RT of the correct button presses and errors made. There are three basic types of errors: incorrect responses, missing responses and commission errors. In a go trial subject can make an incorrect response, i.e., press a wrong button, or miss responding, reflecting lapses in working memory and attention correspondingly. A commission error, i.e., a key press in a No-go trial, reflects failure in response inhibition. Basic errors are summed up as total errors indexing executive function performance in general.

The tests were conducted at the Behavioral Neurology Research Unit, Tampere University Hospital, Finland. The recording room was sound-attenuated, and the ceiling lights of the room were dimmed. The subjects sat at one-meter distance from a 21-inch screen, equipped with a response pad (Cedrus RB-840, Cedrus Corporation, San Pedro, CA, United States) with dedicated keys for each finger. Before starting the test, subjects practiced responding so that they felt confident in executing the test and researcher ensured that the subject learned to do the test. Typically, one practice block was enough to reach confidence. Subjects were instructed to respond as quickly and accurately as possible. The Executive RT Test utilizes Presentation software (Neurobehavioral systems, Inc., Berkeley, CA, United States) to present the stimuli and to register the performance of the subject.

### Behavior Rating Inventory of Executive Function-Adult Version (BRIEF-A)

Behavior Rating Inventory of Executive Function-Adult version is a clinically validated questionnaire of executive function in daily life consisting of nine scales (Inhibit, Self-Monitor, Plan/Organize, Shift, Initiate, Task Monitor, Emotional Control, Working Memory and Organization of Materials) tapping into various parts of executive functioning in daily life. The scales are summarized in two summary indices, Behavioral Regulation Index (BRI) and Metacognition index (MI). BRI is composed of Inhibit, Shift, Emotional Control and Self-Monitor scales while MI is composed of Working Memory, Plan/Organize, Task Monitor and Organization of Materials scales. The Global Executive Composite (GEC) is an overall score that summarizes all the other scores. Three subjective response biases are assessed in BRIEF-A: negativity, inconsistency and infrequency (Roth et al., 2005).

### Statistical Analysis

Changes in RTs were analyzed with repeated measures analysis on variance (ANOVA), where Test and Distractor valence were within subject factors. Errors were analyzed using generalized binary logistic regression as proposed by Jaeger (2008) and Dixon (2008) so that Subject was used as a random effect predictor and Test and Distractor valence as fixed effect predictors. Each error type had its own logistic regression model. If significant interactions were found, data was stratified into groups and groups were analyzed separately.

For RT analysis, only trials with correct response and RT longer than 150 ms were included. For error analysis trial outcome was dichotomized so that for total errors trial outcome was “error” or “correct,” for incorrect responses “incorrect” or “other” (=correct or missing response in Go trial), for missing responses “miss” or “other” (=correct or incorrect response in Go trial) and for commission errors “correct” (=no button press in No-go trial) or “commission error” (=correct or incorrect button press in No-go trial).

Within test learning was studied excluding same blocks one by one from the beginning of both tests and comparing first and second test rounds using the remaining blocks. The excluded blocks could be considered practice blocks.

Test–retest reliabilities were calculated using Pearson’s correlation and ICC with 95 % confident intervals. ICC values were calculated using two-way mixed effects model with single measurements and absolute agreement as suggested by Koo and Li (2016) for test–retest situations. Two-way mixed effects model is identical to the Shrout and Fleiss ICC (2,1) model (Shrout and Fleiss, 1979). The model accounts for both systematic and random errors in test–retest calculation. A systematic change, like improvement due to practice, weakens ICC unlike Pearson’s correlation coefficient which accounts only for random error. ICC coefficient and Pearson’s correlation coefficient values were calculated for RTs (subject’s mean RT), errors (error percentage) and BRIEF-A major indices (T-scores).

After the initial analysis we also studied the impact of age on learning. For this analysis data was stratified into two groups by age. In the first group (*n* = 11, mean 27.0y, min 21y, max 32y, SD 3.8y) were subjects younger than 40 years old and in the second group subjects 40 years old or older (*n* = 9, mean 49.4y, min 41y, max 60y, SD 5.6y). New analysis with the age grouping were executed in which Age group was a between groups factor in ANOVA and a fixed effect predictor in binary logistic regression.

Two subjects were excluded as outliers. One subject was excluded from the Executive RT Test performance analysis because of unusually high amount of commission errors and misses in the first test which indicates a misunderstanding of the task rule. Subject’s total error rate was more than three SDs from the overall mean. One subject was removed from the BRIEF-A analysis because of identical, lowest possible scores in both tests, classified by the validity check of BRIEF-A as “infrequent” (Roth et al., 2005). Both outliers were excluded from the Executive RT Test–BRIEF-A correlation analysis.

BRIEF-A indices between the two tests were compared using Wilcoxon signed rank test. Correlation between mean BRIEF-A scores and the errors and reactions times were calculated using Spearman’s rank correlation. For the correlation analysis the Executive RT Test results and the BRIEF-A scores from test 1 and test 2 were pooled together. The Executive RT test results were pooled by excluding Test factor from ANOVA and the BRIEF-A scores were pooled by establishing means across the two tests.

All statistical analysis was conducted with R 3.3.2 (R Core Team, 2016) using ez package v.4.4-0 (Lawrence, 2016) for repeated measures ANOVA, lme4 package v.1.1-12 (Bates et al., 2015) for regression analysis and psych package v.1.7.3 (Revelle, 2017) for ICC.

## Results

### Performance in Repeated Assessment With Executive RT Test

When the entire test was analyzed, logistic regression showed a statistically significant decrease in incorrect button responses in the second test (Table 1). The odds for incorrect responses in the second test was 64% lower than in the first test (OR 0.36, 95% CI 0.19-0.68). Median incorrect response rate in the first test was 0.78% (interquartile range 1.17%- points) and in the second test 0.39% (IQR 0.78%- points). Analysis of the other error types (Total errors, missing responses and commission errors) did not reveal statistically significant differences between the two tests.

**Table 1:** The median error rate percentages with interquartile ranges classified by the error type (complete test).

	Median error percentage (Q1–Q3)		
		
	Test 1	Test 2	OR (95% CI)
***Executive RT Test***			
* Total errors*	1.56 (0.78–3.32)	1.34 (0.98–1.96)	0.72 (0.47–1.10)
*Incorrect responses*	0.78 (0.39–1.56)	0.39 (0.00–0.78)	0.36 (0.19–0.68)^∗^
*Commission errors*	0.39 (0.00–1.37)	0.78 (0.39–1.17)	1.21 (0.61–2.38)
*Missed responses*	0.00 (0.00–0.20)	0.00 (0.00–0.20)	3.02 (0.61–15.00)


*Subjects’ probability to respond incorrectly decreased by 64% in the second test. ^∗^Statistically significant difference.*

**FIGURE 2:**
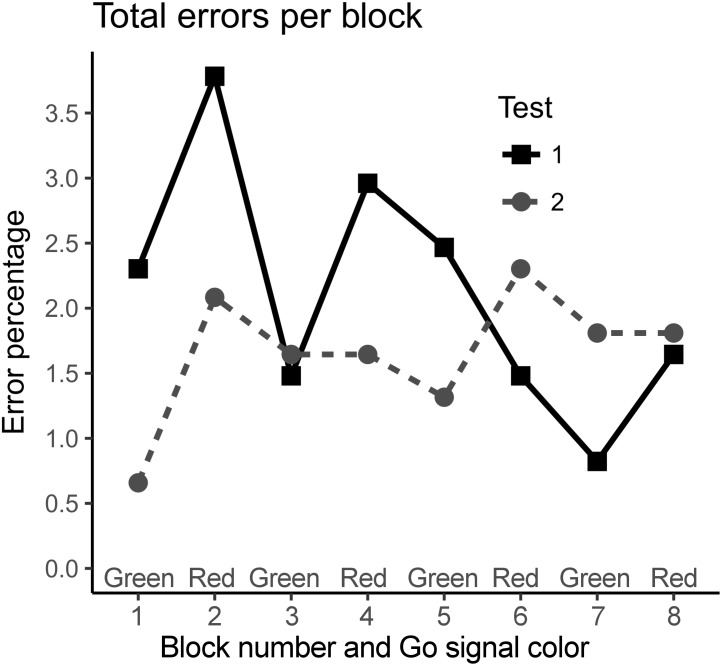
The total error percentages of the Go trials per block in the first test (Test 1) and in the retest (Test 2). The rate of total errors decreased towards the end of the first test but within the second test the error rate remained stable.

**FIGURE 3:**
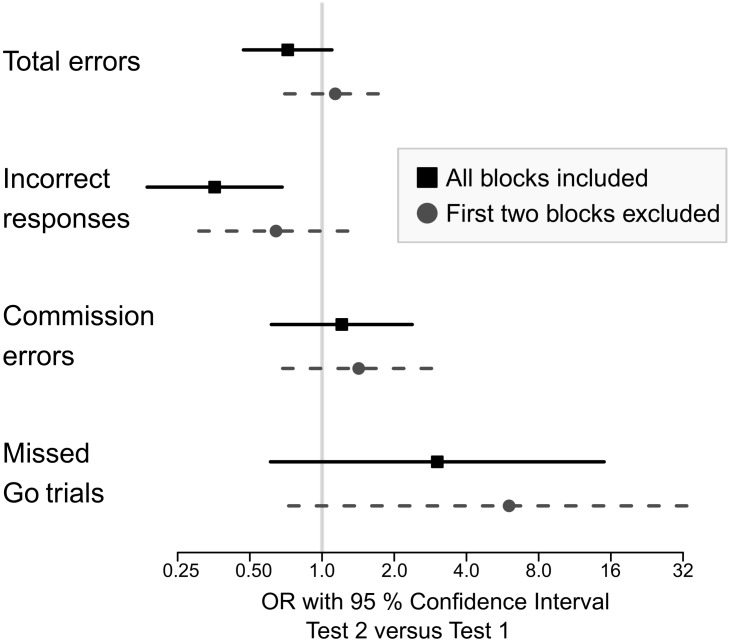
Odd’s ratio with 95% confidence interval for making an error when all the blocks were included and when the two first blocks were excluded. When all blocks were included, there was a significant difference in the probability for responding incorrectly. When the first two blocks were removed, the difference disappeared.

When within test learning was studied, during the first test the amount of total errors and incorrect responses decreased toward the end of the test, but during the second test the error rates stayed stable throughout the test (Figure 2). When blocks were excluded from the beginning of both tests one by one, after the removal of the first two blocks (25% of the total number of eight blocks, one block = 64 single trials) there was no longer any difference in the odds of any error types (Figure 3).

Re-analysis with the Age group as a fixed effect predictor resulted in significant decrease in total errors (OR 0.48, 95% CI 0.25-0.93) in addition to the decrease in incorrect responses. Similar to the model without the age group, the difference in incorrect responses and total errors disappeared for both age groups after the first two blocks were excluded from the analysis. Age did not affect to odds of subject’s errors.

**FIGURE 4:**
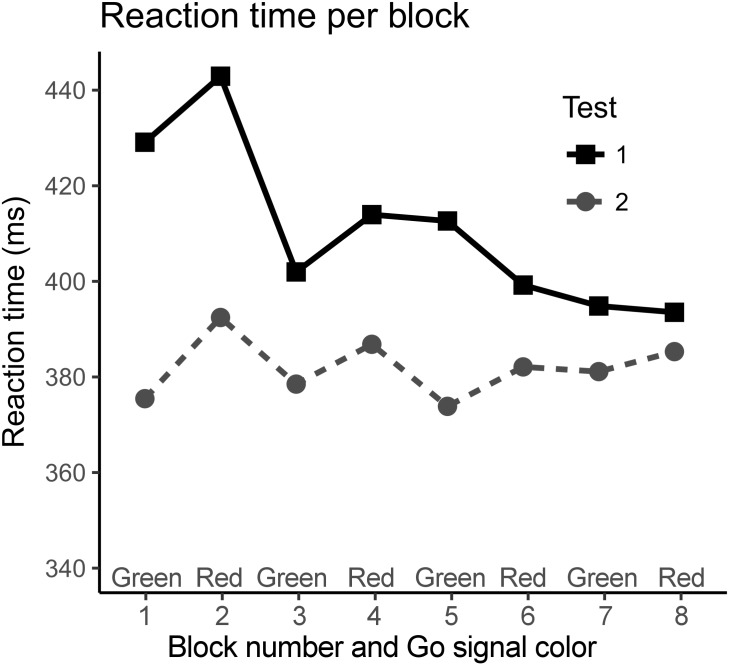
The average reaction times per block in the first and in the second test. When only the last four blocks were compared, there was no significant difference in reaction times. The curve of the first test has a decreasing trend but in the second testing session reaction times remained stable throughout the test.

When the entire test was analyzed, RTs improved from 411 (SD 74) ms in the first test to 383 (69) ms in the second test (Table 2) and repeated measures ANOVA revealed improvement to be statistically significant [*F*(1,17) = 14.43, *p* = 0.001]. Like in errors, there was a decreasing trend in RTs in the first test, but in the second test RTs remained stable throughout the test (Figure 4). When test blocks were excluded one by one from the beginning of the test, the difference in RT between the two tests disappeared after the exclusion of first four blocks (50% of the blocks).

**Table 2:** Reaction times with standard deviations.

	*Test 1*			*Test 2*		
		
	All	Young	Older	All	Young	Older
***Executive RT Test***						
* Task reaction time*	411 (74)	387 (72)	444 (68)	383 (69)	365 (73)	406 (58)
*Task reaction time with emotional distractor*	413 (74)	391 (74)	444 (68)	381 (68)	362 (69)	406 (62)
*Task reaction time with neutral distractor*	409 (75)	383 (71)	445 (68)	384 (70)	368 (78)	407 (54)


### Impact of Emotional Distractors on Performance

**FIGURE 5:**
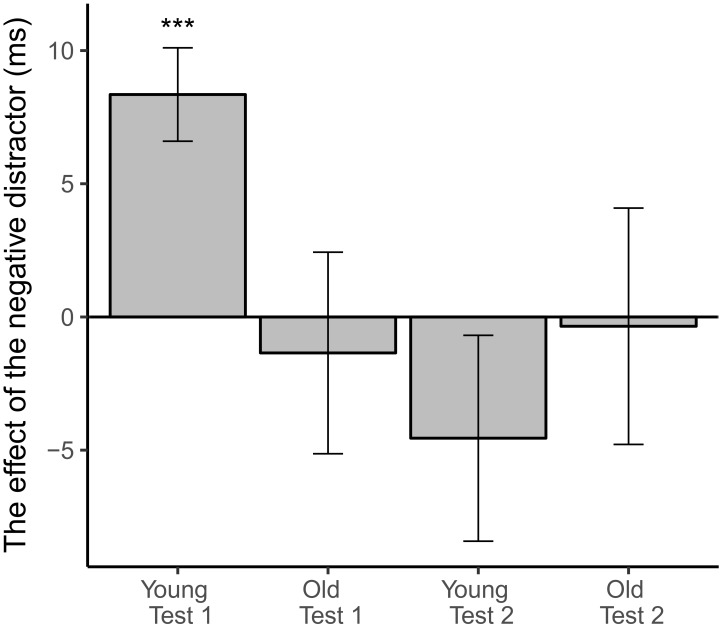
The difference in task reaction times when negative distractor (gray bars) was presented in contrast to when neutral distractor was presented (baseline). Error bars represent standard error of the mean difference. Emotionally negative distractor slowed reaction times in young subjects in the first test. ^∗∗∗^Statistically significant difference (*p* < 0.001).

There was no main effect of emotion for RTs [*F*(1,17) = 0.17, *p* = 0.68] and no impact of emotion on the odds of errors. In the RT analysis Test and Emotion interaction was statistically significant [*F*(1,17) = 4.39, *p* = 0.05] as well as interaction of Age, Test, and Emotion close to significance [*F*(1,17) = 4.16, *p* = 0.057]. Based on these interactions and the previous literature on the effects of threat-related distractors on performance in the Executive RT test (Hartikainen et al., 2012), *post-hoc* analyses were performed. In the test 1 Age and Emotion interaction was statistically significant [F(1,17) = 6.51, *p* = 0.021]. *Post-hoc* analysis revealed that emotional distractor slowed down mean RTs in young subject group in comparison to emotionally neutral distractor in the first test [F(1,10) = 22.72, *p* = 0.001] but not in the older subjects [*F*(1,7) = 0.13, *p* = 0.73, Table 2 and Figure 5]. The emotional distractor did not affect either group in the retest session (Table 2 and Figure 5).

### Correlations Between Performance in Executive RT Test and BRIEF-A Scores

Average RTs in the Executive RT Test correlated positively with GEC score derived from overall BRIEF T-scores (rho = 0.56, *p* = 0.02) and with the BRI (rho = 0.58, *p* = 0.01). The MI did not correlate with the RTs. Three individual scales correlated with RTs: Shift (rho = 0.54, *p* = 0.02), Self-Monitor (rho = 0.57, *p* = 0.01), Initiate (rho = 0.66, *p* < 0.01). The Inhibit scale correlated almost significantly with the RTs (rho 0.45, *p* = 0.06). None of the error types correlated with the BRIEF-A summary indices.

### Test–Retest Reliability

Intra-class correlation under 0.5 can be classified as a poor, 0.5–0.75 moderate, 0.75–0.90 good and above that as an excellent reliability (Portney and Watkins, 2009). Task RTs showed good reliability in the Executive RT Test and ICC for the total errors and the incorrect responses were in the upper section of the moderate range of reliability (Table 3).

**Table 3:** The test–retest ICC and Pearson’s correlation coefficients with 95% confidence intervals.

	ICC	95% CI	Pearson	95% CI
***Executive RT Test***				
*Reaction time*	0.83	0.39–0.94	0.89	0.74–0.96
*Total errors*	0.72	0.41–0.88	0.8	0.54–0.91
*Incorrect responses*	0.68	0.24–0.87	0.8	0.55–0.92
*Commission errors*	0.25	-0.23–0.63	0.25	-0.23–0.63
*Missed Go trials*	-0.14	-0.57–0.34	0.25	-0.57–0.32
***BRIEF-A***				
*BRI*	0.78	0.53–9.91	0.8	0.54–0.92
*MI*	0.66	0.30–0.85	0.67	0.30–0.86
*GEC*	0.86	0.67–0.94	0.87	0.68–0.95


*ICC < 0.5 can be classified as a poor, 0.5–0.75 as moderate, 0.75–0.90 as good, and >0.90 as excellent reliability. The test retest reliability of the Executive RT test ranged from moderate to good. The number of the commission errors and the missed Go trials was too low to calculate reliable coefficient for these error types.*

There were no statistically significant changes in the scores of the BRIEF-A self-report questionnaires but there was a tendency toward lower scores in the second test in both the overall score and in the individual indices. The mean T-score of the overall index, the GEC, decreased from 47.26 (SD = 7.42) to 45.89 (SD = 7.71), Wilcoxon signed ranks test *Z* = -1.910, *p* = 0.056.

Test–retest correlation coefficients for commission errors and missed trials were not statistically significant. The test–retest reliability of the BRI was moderate compared to test–retest reliability of the MI and the GEC. Pearson’s correlation coefficients were higher than the ICCs in most categories.

## Discussion

The Executive RT Test showed promise as a sensitive and reliable test of higher cognitive control functions reflecting brain health. RTs in the Executive RT Test was shown to correlate with subjective assessment of executive functions in daily life and the test–retest reliability was competitive to conventional neuropsychological tests of executive functions. Like in all tests of executive functions, there was a practice effect in the Executive RT Test. However, learning occurred during the first blocks of the first test and after that performance remained stable. Thus, removing the first few blocks from the analysis allows one to compare performance at different time points without practice effect confounding the results. In addition to potentially reflecting subjective challenge in executive functions in everyday life, the Executive RT Test was sensitive enough to show that a threat related distractor slowed performance speed in young adults in the first test. However, young adults adapted to the threat related distractor and after adaptation the effect could not be seen in the second test. We speculate that lack of adaptation to threat related emotional stimuli in young subjects could reflect deviation from healthy emotion–attention interaction. In conclusion, the Executive RT Test holds promise as a potential indicator of brain health.

It is remarkable that RTs in the Executive RT test correlated with individual’s self-assessed executive function performance in daily life in general and more specifically, with the composite score reflecting behavioral regulation. These results are in line with previous report on complex processing speed measures correlating significantly with executive control (Cepeda et al., 2013). The current results are even more remarkable considering the subjects were healthy and the differences in the BRIEF-A scores subtle. Earlier studies have found that BRIEF-A scores do not correlate with performance in executive function tasks (Rabin et al., 2006) and subjective challenges reported in BRIEF-A by patients with focal brain lesion are not necessarily detected in neuropsychological tests (Løvstad et al., 2012). It is a major clinical challenge that objective evidence for executive dysfunction is frequently lacking even though subjects experience challenges in executive functions in their daily life. To that end, in addition to scientific relevance, the current results bear clinical relevance in suggesting that RTs in an integrated test of executive functions correlate with subjective evaluations of executive functions. The correlation between the Executive RT Test and BRIEF-A validates the Executive RT Test as a measure of global executive functions.

When the test–retest reliability of commonly used neuropsychological tests are compared to the test–retest reliability of the Executive RT Test, the Executive RT Test is competitive in repeated assessment of executive functions. Lowe and Rabbitt (1998) and Lemay et al. (2004) have studied test–retest reliabilities of commonly used neuropsychological tests in healthy middle-aged or elderly subjects who have executed the tests twice, 2–4 weeks apart. Both of those studies showed that the test–retest reliability is better if the assessment is based on time measurements but reliability weakens if it is based on accuracy measurements (Lowe and Rabbitt, 1998; Lemay et al., 2004). In those studies Tower of London test had poor reliability, less than 0.50, if the analysis was based on the number of moves and better (0.45–0.83) if the variable was time. Also, the test–retest correlation coefficients of Stroop test were 0.80 and 0.53, when based on completion time and less than 0.5 when based on errors. Similarly, the Executive RT Test showed better test–retest correlation for RTs (ICC coefficient 0.83) than accuracy measures such as total errors (ICC 0.72) or incorrect responses (ICC 0.68). Especially tests requiring set shifting have poor reliability. In the study by Lemay et al. (2004) for an intra-dimensional and extra-dimensional set shifting task, a computerized analog of Wisconsin Card Sorting test, the correlation coefficient was only 0.09 for intra-dimensional rule change errors and 0.70 for extra-dimensional rule change errors. In the same study, a concept shift test, similar with conventional Trail Making Test, had a poor ICC ranging from 0.06 to 0.16 even if the measured variable was task completion time.

Practice effect in repeated RT and executive function tests is a major challenge when assessing for example the impact of clinical interventions on brain health and efficiency of executive functions. Practice effect is especially significant in traditional pen and paper tests and their computerized versions, such as Stroop Test, Wisconsin Card Sorting Test and Trail Making Test, but impacts also computer-based tests (Lowe and Rabbitt, 1998; Lemay et al., 2004; Bartels et al., 2010).

Earlier research on computer based tests suggests that in repeated testing of executive functions with 2 to 3-week interval in between the tests the practice effect is the largest from the first test to the second test and plateaus after that (Bartels et al., 2010). However, there is only little knowledge about the time course of the practice effect in these tests. Does the improvement occur within the tests or between tests? In the current study the practice effect occurred during the first few blocks of the first test. The performance after the initial learning was stable and there was hardly any change in the performance between the latter part of the first test and at the beginning of the second test. When practice effect is known and controlled for, sensitive computerized tests of executive function such as the Executive RT Test, can be used in intervention studies including cardiac operations (Liimatainen et al., 2016), anesthesia methods that may influence brain health and treatments targeting the brain such as neuromodulation (Hartikainen et al., 2014), to measure the impact of the intervention in question on the efficacy of higher cognitive functions reflecting brain health in general. This will allow research efforts that provide the basis for development of variety of treatments and interventions to be geared toward optimal brain health.

While there were no differences in the average RTs or error rates between the age groups, the negative emotional distractor prolonged RTs of younger participants in the first test but had no effect in older participants. The negative distractor did not have any effect in the second test in either group. The emotional interference of RTs in younger participants in the first test is in line with earlier studies showing emotional stimuli prolonging RTs in young adults (Hartikainen et al., 2000) and in teenagers (Ramos-Loyo et al., 2017). In our previous study where a prior version of the Executive RT Test with similar distractors was used, threat related distractors impaired response inhibition in young healthy subjects (Hartikainen et al., 2012). The reason for the difference observed in the first test in emotional interference between the age groups is unclear, but there are studies suggesting that aging may alter reactions to negative stimuli. Kaszniak and Menchola (2012) propose that when people age, their responses to negative stimuli may weaken (Kaszniak and Menchola, 2012). Scheibe et al. (2015), on the other hand, have suggested that aging may alter emotion regulation strategies so that older subjects have a tendency to direct their attention away from negative emotional stimuli, while younger subjects tend to predominantly use reappraisal for emotion regulation (Scheibe et al., 2015). Attentional mechanisms are faster strategies compared to re-appraisal, as they occur earlier in the emotion regulation process (Webb et al., 2012). In the second test no emotional interference was observed in either age group. The change in younger age group could be explained by change to a faster emotion regulation strategy, such as attentional mechanisms suggested to be used by older individuals, or by habituation to emotional distractor so that no top down emotional control was needed any more (Bordi and LeDoux, 1992; Breiter et al., 1996).

Changes in reactions and in adaption curves to emotional stimuli could be used to objectively assess affective dysfunction in brain disorders, especially when combined with EEG and ERP (Event Related Potentials). For example, Mäki-Marttunen et al. (2015) and Sun et al. (2015) have demonstrated with a similar computerized test and ERP responses enhanced attention capture by threat-related emotional stimuli in clinical populations with predisposition to depressive symptoms such as subjects with history of mild head injury and patients treated with DBS targeted at anterior thalamus due to refractory epilepsy, correspondingly (Mäki-Marttunen et al., 2015; Sun et al., 2015). These findings are in line with attention bias to as well as increased neural activity to negative emotional stimuli in depression (Leppänen, 2006). To that end, we speculate that assessing emotional interference on performance and the impact of repeated exposure to threat stimuli might offer a way to get objective evidence for alterations in emotion–attention interaction in affective disorders.

The most notable weakness of the Executive RT Test was somewhat low error rate resulting in large confidence intervals in the logistic regression and making it impossible to calculate reliable test–retest correlation coefficients for commission errors and missed responses. Thus, an alternative explanation for commission errors and missed trials not improving may reflect a ceiling effect. Despite the fact that the stimuli were presented rapidly, many cognitive processes were engaged simultaneously and that initially many subjects perceived the test as difficult, the test seems to be too easy for healthy subjects. The low rate of the commission errors complicates the assessment of response inhibition and limits how broadly it represents different aspects of executive functions in healthy subjects. However, even though there was a ceiling effect with healthy subjects in this study, in a study by Liimatainen et al. (2016) patients with aortic stenosis tested before and after aortic valve replacement surgery have shown improved performance post-operatively specifically for commission errors and missed responses (Liimatainen et al., 2016). Thus, ceiling effect may occur only in healthy participants with intact or high cognitive abilities and not in clinical populations with compromised cognitive performance. Another weakness in the current study is the small sample size limiting the generalizability of the results. Furthermore, the study was conducted in a healthy population and in order to extrapolate the relevance of the current findings to clinical populations, future studies on them are called for. However, currently our previous studies on mild head injury (Hartikainen et al., 2010), patients with focal lesion to OFC (Mäki-Marttunen et al., 2015, 2017; Kuusinen et al., 2018) and patients with DBS treatment for refractory epilepsy (Hartikainen et al., 2014) provide some support that Executive RT Test is also sensitive in detecting executive dysfunction in clinical populations.

Efficient executive functions depend on intact frontal networks extending throughout the brain, which can be impaired by brain disorders, brain injury or treatments that impact the brain as well as various other factors affecting brain health in general. In the current clinical practice the traditional neuropsychological tests tend to be insensitive to subtle changes in executive functions and unsuitable for repeated testing. Furthermore, the currently used neuropsychological tests frequently fail to detect the subjective challenges the patients experience in executive functions. To that end, the Executive RT Test shows promise as a sensitive test of executive functions with RTs correlating with subjective challenges, with performance measures being resistant to practice-effect after sufficient amount of practice and showing good test–retest correlation. There is a call for a valid, sensitive, reliable and repeatable test of executive functions. The Executive RT Test, that allows measurement of RTs in a task that challenges multiple executive functions simultaneously and in context of emotional distractors is a good candidate to fill this void and further, shows potential as a more general indicator of brain health.

## Data Availability Statement

The datasets are available on request.

## Author Contributions

ME participated in the data collection, conducted the data analysis and contributed to the writing of the article. JP was involved in the data collection, the statistical analysis and the writing of the article. KMH contributed to the experimental design, the supervision of the data analysis and to the writing of the article.

## Conflict of Interest Statement

The authors declare that the research was conducted in the absence of any commercial or financial relationships that could be construed as a potential conflict of interest.
